# High-yield scalable graphene nanosheet production from compressed graphite using electrochemical exfoliation

**DOI:** 10.1038/s41598-018-32741-3

**Published:** 2018-09-28

**Authors:** Thomas C. Achee, Wanmei Sun, Joshua T. Hope, Samuel G. Quitzau, Charles Brandon Sweeney, Smit A. Shah, Touseef Habib, Micah J. Green

**Affiliations:** 1 Artie McFerrin Department of Chemical Engineering, Texas A&M University, College Station, TX USA; 2 Department of Materials Science and Engineering, Texas A&M University, College Station, TX USA

## Abstract

Electrochemical exfoliation is a promising bulk method for producing graphene from graphite; in this method, an applied voltage drives ionic species to intercalate into graphite where they form gaseous species that expand and exfoliate individual graphene sheets. However, a number of obstacles have prevented this approach from becoming a feasible production route; the disintegration of the graphite electrode as the method progresses is the chief difficulty. Here we show that if graphite powders are contained and compressed within a permeable and expandable containment system, the graphite powders can be continuously intercalated, expanded, and exfoliated to produce graphene. Our data indicate both high yield (65%) and extraordinarily large lateral size (>30 μm) in the as-produced graphene. We also show that this process is scalable and that graphene yield efficiency depends solely on reactor geometry, graphite compression, and electrolyte transport.

## Introduction

Graphene, i.e., a single layer of sp^2^-hybridized carbon, is rightly touted for its superlative material properties and has been demonstrated both as a novel building block and as an additive for a wide range of multifunctional materials^1,2^. Such materials are used for energy storage, structural composites, and advanced electronics^3–9^. Industry reports estimate that graphene demand will reach over 4100 tons/year by 2026^10^.

A variety of graphene family materials are a major target for industrial scaleup as high-value-add fillers and additives^11^. Although a wide range of graphene production methods have been studied, the difficulty of producing large quantities of graphene from flake graphite has been a persistent problem. Although bottom-up methods of growing graphene sheets on substrates through vapor deposition have been successfully applied in industry, these techniques are limited to large-area graphene but cannot produce large masses of graphene^12,13^. The most commonly used top-down methods for producing graphene from graphite suffer from a longstanding quality vs. quantity tradeoff^14^. There are two large-quantity methods of producing graphene from graphite: (i) The oft-used modified Hummers’ method involves extensive oxidation^15,16^, but the resulting graphene oxide (GO) nanosheets are defect-laden and electrically insulating. Graphene oxide may be thermally or chemically reduced to yield “reduced graphene oxide” (rGO) nanosheets that retain some of their defects and oxygen-containing functional groups^17–20^. (ii) In contrast, pristine graphene is typically mechanically exfoliated from graphite, often in a liquid medium with sonication or shear mixing, but the actual product is a mixture of pristine graphene and unexfoliated graphite^16,21–31^. Pristine graphene production has been plagued with difficulties either in production rate or in the number of layers present in each graphene platelet. This has led to much confusion in metrics and nomenclature; both “graphene nanoplatelets” and “nanographene platelets” are commercially available (both many-layers). Several recent reports have described advances in scalable (better than linear) methods for production of few-layer pristine graphene from graphite^21^. However, this analysis invariably neglects the problem of separating the as-produced graphene from the unexfoliated graphite; such separation techniques are notoriously difficult to scale up^32^. If high-yield production methods can be attained, the separation problem becomes far less pressing.

## Prior Work in Electrochemical Exfoliation

**Figure 1:**
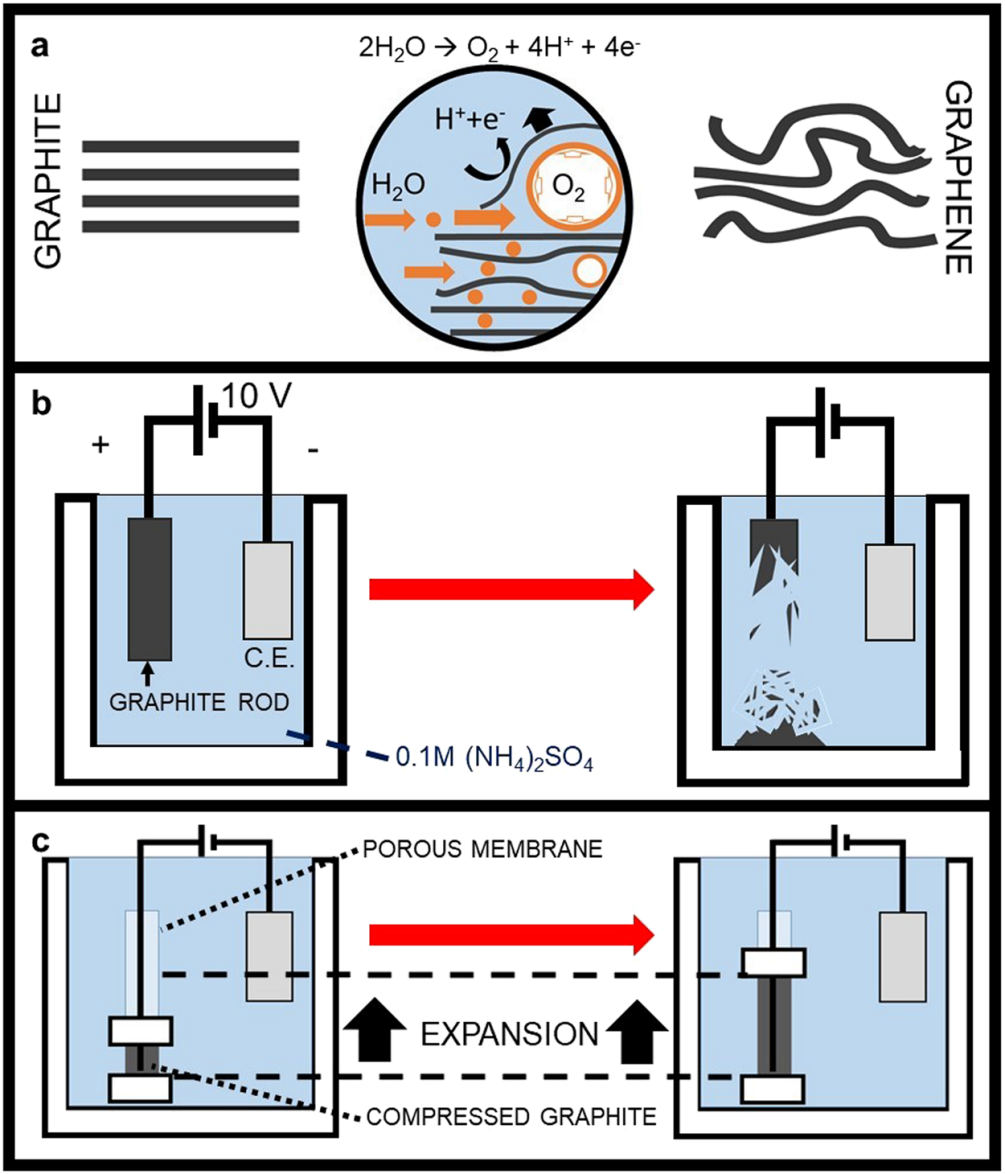
(**a**) Mechanism of electrochemical exfoliation: Graphite evolves to graphene as sulfate intercalation and oxidation proceeds. (**b**) Prior efforts to use this concept have always resulted in the graphite working electrode disintegrating during intercalation (C.E. denotes the counter electrode). (**c**) Schematic illustration of our process for electrochemical exfoliation of graphite flakes in a permeable, expandable container.

Electrochemical exfoliation of graphene is an alternative to the mechanical or oxidation-driven options discussed above for bulk production of graphene from parent graphite^33–35^. An applied voltage drives the ionic species in an electrolyte to intercalate into the graphite electrode and increase the inter-layer distance^36^. For example, in ammonium sulfate, the sulfate ions and water molecules migrate into the interstitial regions of the graphite and locally form gas bubbles (such as SO_2_, O_2_), which forces adjacent sheets apart (Fig. 1a)^1^.

Despite these advances in electrochemical exfoliation, there are a number of major challenges facing the field: (i) Only graphite monoliths (as opposed to loose graphite powders) may be used as a source for electrochemical exfoliation because the graphite electrode must be continuous, electrically conductive, and connected to the external power supply. (ii) Most importantly, if electrochemical exfoliation actually occurs and the monolith is expanded to form graphene, then the monolith disintegrates and the electrochemical exfoliation process stops. The graphite electrode tends to fall apart as intercalation proceeds, and when graphite pieces become disconnected from the monolith (and thus, the circuit), they can no longer participate in the intercalation process. The degradation (Fig. 1b) of the graphite rod results in a low yield of graphene and requires further separation of unexfoliated material.

Liu *et al*. attempted to address these challenges by placing the monolithic graphite electrode at the bottom of the exfoliation vessel, with the expectation that gravity would allow the disintegrated graphite particles from the monolith to remain connected by gravity, even as exfoliation progresses^34^; they reported a slight improvement in yield (based on sonicated dispersions in dimethylformamide), but graphitic particle retention is uncontrolled. Abdelkader *et al*. proposed (but have not attempted) a similar setup where a steady flow of electrolyte solution is continuously fed through the setup, allowing any exfoliated, floating graphene to be collected^37^. Wang *et al*. demonstrated that paraffin coating outside the graphite electrode could effectively confine the electrochemical exfoliation to a defined volume^38^. However, intensive sonication in N-Methyl-2-pyrrolidone and further centrifugation were used as post-processing in this case. We discuss the “yield” of these processes in further detail below.

## Results

### Compressed, expandable electrodes

Our method overcomes these challenges and provides a new approach for scalable, high-yield graphene production with large sheet size^39^. As shown in Fig. 1c, rather than a monolithic graphite rod electrode, we utilize graphite flakes without any binder inside a permeable container as the working electrode. Graphite flakes are pressed together by a movable clip on the top to form an electrically conductive electrode. To connect the graphite flakes with the power supply, a piece of platinum wire (current collector) is inserted in the permeable container. This ensures the electrical connection between the graphite flakes and the external pressure created by the clips. A piece of graphite foil works as a counter electrode. The working electrode and the counter electrode are both immersed in an aqueous electrolyte (0.1 M (NH_4_)_2_SO_4_). When a positive voltage (+10 V) is applied to the working electrode, the graphite flakes are continuously intercalated and expanded.

Because of the compression of the container, the graphite flakes remain in electrical contact, even as they expand due to gas evolution between atomic layers of graphite flakes. (Note: A simple experiment demonstrates that external pressure is necessary to maintain conductivity throughout the graphite structure, Fig. S1.) The mechanism is the same as the prior work on monolithic graphite rods: the applied voltage drives the reduction of water at the cathode and the hydroxyl ions (OH^−^) attack graphite at the edges and grain boundaries^40,41^; the oxidation at the edges and grain boundaries expands the graphite, facilitating the intercalation of sulfate ions (SO_4_^2−^); the reduction of SO_4_^2−^ anions and oxidation of water generate various gases, such as SO_2_, O_2_, and others. The expanding gases separate the graphene layers, resulting in electrochemically exfoliated graphene (EEG). The counter electrode does not experience any exfoliation. This method is suited to a range of precursor graphitic materials, unlike prior electrochemical methods that require a monolithic graphite structure.

The wetting of the graphite by the electrolyte may be hindered by graphite’s natural hydrophobicity since the reaction involves oxidation only at wetted graphite surface sites. We used HNO_3_ to convert hydrophobic graphite to hydrophilic graphite by adding oxygen-containing groups to any pre-existing defect sites (Fig. S2**)**^42^. This improved wetting leads to improved ionic transport.

In our batch reactor schematically depicted in Fig. 1c, we introduce graphite flakes (pre-treated as described above) into a tubular dialysis membrane (as the permeable container) and sealed it with two clips (the one on the top is movable). The graphite flakes are electrochemically expanded using the procedure described above. After the process is complete, the resulting product is washed with DI water to remove the electrolyte. We then suspend the product in a vessel filled with DI water where any unexfoliated material rapidly sediments (within 10 minutes). The supernatant from this vessel is collected, and the suspended graphene (the suspension is termed as “EEG-1”) in the supernatant is collected as product. The yield of the process is defined by the ratio of EEG-1 mass to the starting graphite mass. We confirm that this EEG-1 is graphene (distinct from the parent graphite) in more detail below. The EEG-1 may be directly freeze-dried to form graphene powder (the powder is termed as “EEG-2”), or it may be resuspended in DI water and shear mixed (the suspension after shear mixing is termed as “EEG-3”) prior to freeze drying (the freeze-dried powder is termed as “EEG-4”) or vacuum filtration to form a buckypaper (the buckypaper is termed EEG-5) (Fig. S3**)**. (Shear mixing time and speed were varied and are discussed below).

### Characterization of as-produced graphene

**Figure 2:**
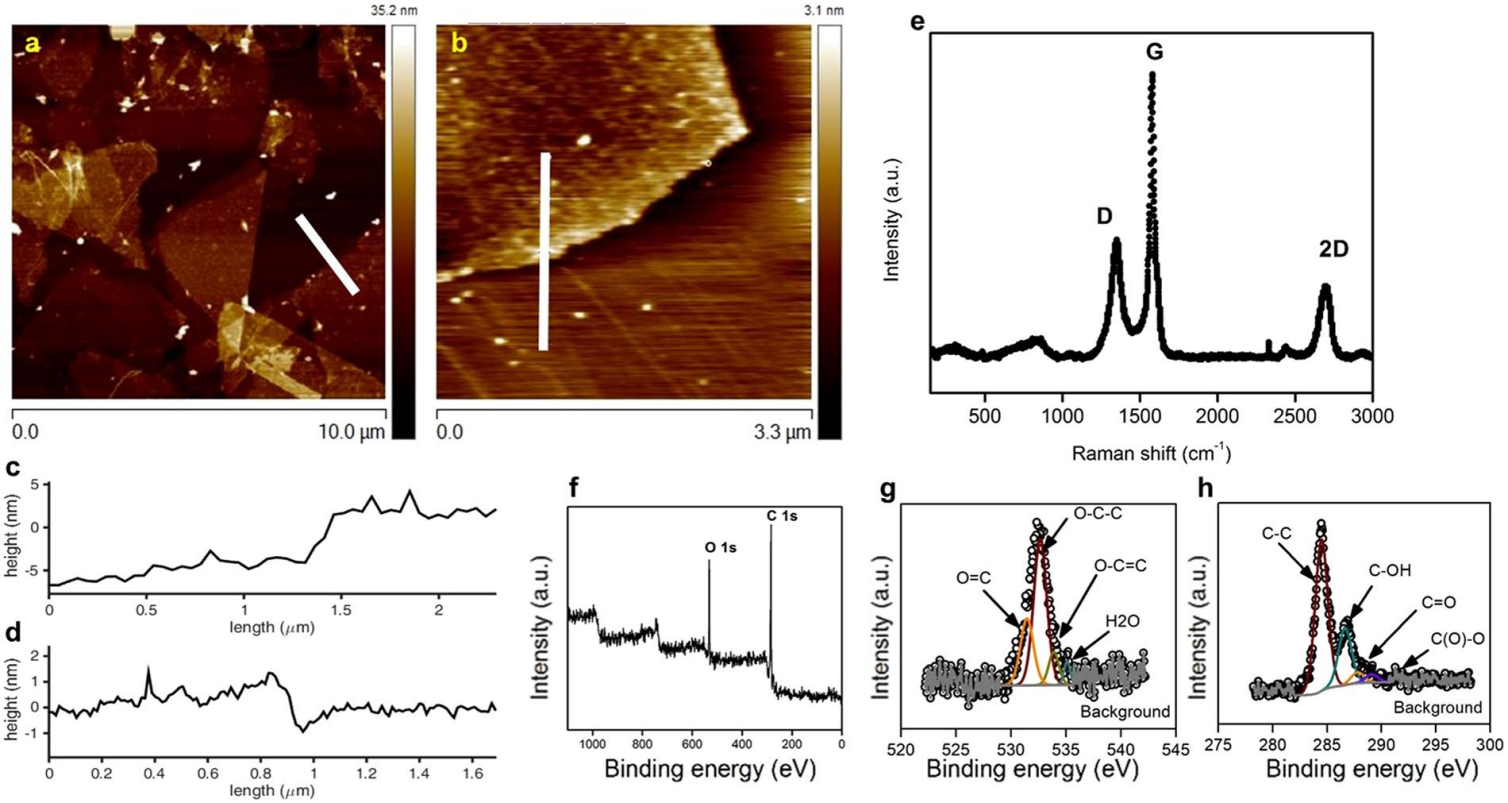
(**a**,**b**) The AFM height map of EEG-3 deposited on a mica substrate show that the material has a thickness less than 10 nm. (**c**) and (**d**) show the height profiles of the lines in (**a**) and (**b**), respectively. (**e**) The Raman spectrum of EEG-2 shows a 2D peak characteristic of graphene. (**f**) X-ray photoelectron spectra (XPS) of EEG-2 with (**g**) deconvoluted O 1s and (**h**) C 1s peaks shows the C/O ratio of the EEG-2.

We utilized atomic force microscopy (AFM) to study the sheet size and thickness of EEG-3. Water-suspended EEG-3 (after electrochemical exfoliation for 4 hours and shear mixing at 8000 rpm, 1 hour) was drop-casted on mica directly for AFM (Fig. 2a,b). The height profile indicates few-layered graphite nanosheets with a thickness between 2–7 nm (Fig. 2c,d). We also used Raman spectroscopy to characterize the structure of EEG-2 and EEG-4 (Fig. 2e). We performed Raman spectroscopy with a 532-nm excitation laser on EEG-2 and EEG-4 pressed on glass slides. The typical Raman spectrum of graphene displays a D peak at 1350 cm^−1^, a broad G peak at 1580 cm^−1^, and 2D peak at 2680 cm^−1^. The shape of the 2D peak identifies the number of layers in graphene^43^. The G peak corresponds to the first-order scattering of the E_2g_ mode of sp^2^ carbon atoms. The prominent D peak is from the breathing mode of the sp^2^ carbon atoms and activated by the existence of defects, including edges, functional groups (hydroxyl and epoxide groups), and structural disorders. The shape of the 2D peak identifies the number of layers in graphene^43^. The ratio of D peak to G peak (I_D_/I_G_) varies from 0.9 to 1.2 in different spots. Typically, an I_D_/I_G_ ratio of 1.2 is very similar to chemically or thermally reduced GO (~1.1–1.5)^44^. The symmetric 2D peak of our EEG-2 is quite similar to previously reported few-layered graphene. Note that the shear mixing itself does not appreciably alter the Raman signature of the material **(**Fig. S4**)**. X-ray photoelectron spectroscopy (XPS) was used to probe the chemical composition of the EEG-2 (Fig. 2f–h) (no shear mixing). The EEG-2 shows 16.7 atom % oxygen content. The corresponding C/O ratio of 4.98 for EEG-2 is significantly lower than prior work on electrochemically exfoliated graphene, suggesting a higher degree of oxidation of the parent graphite during the electrochemical exfoliation process^1^. This occurs because our process is not arrested by the disintegration of the graphite electrode, in contrast to prior studies. The EEG-2 shows less oxygen functional groups than GO from Hummers’ method, but more oxygen functional groups than thoroughly thermal-reduced graphene oxide. This means our EEG-2 has similar chemical/surface properties to partially reduced graphene oxide. Thermogravimetric analysis (TGA) analysis reveals a similar trend (Fig. S5).

To measure the electrical conductivity of the as-prepared EEG-3, we prepared buckypaper via vacuum filtration of the EEG-3 (8000 rpm, 1 hour shear mixing) and this buckypaper is termed as “EEG-5”. This buckypaper showed an electrical conductivity of 2.1 ± 0.2 × 10^3^ S/m, an excellent value similar to that of reduced graphene oxide, demonstrating that the EEG-5 is conductive at levels comparable to prior methods (Fig. S6). We also demonstrate that EEG-4 can be used as a conductive filler in polymer composites. We fabricated a 5 wt.% EEG-4 and polycarbonate (PC) composite using solution-casting and hot-pressing; the resulting electrical conductivity is 2.0 S/m, which is remarkably high compared against other graphene/polymer composites (Fig. S7).

**Figure 3:**
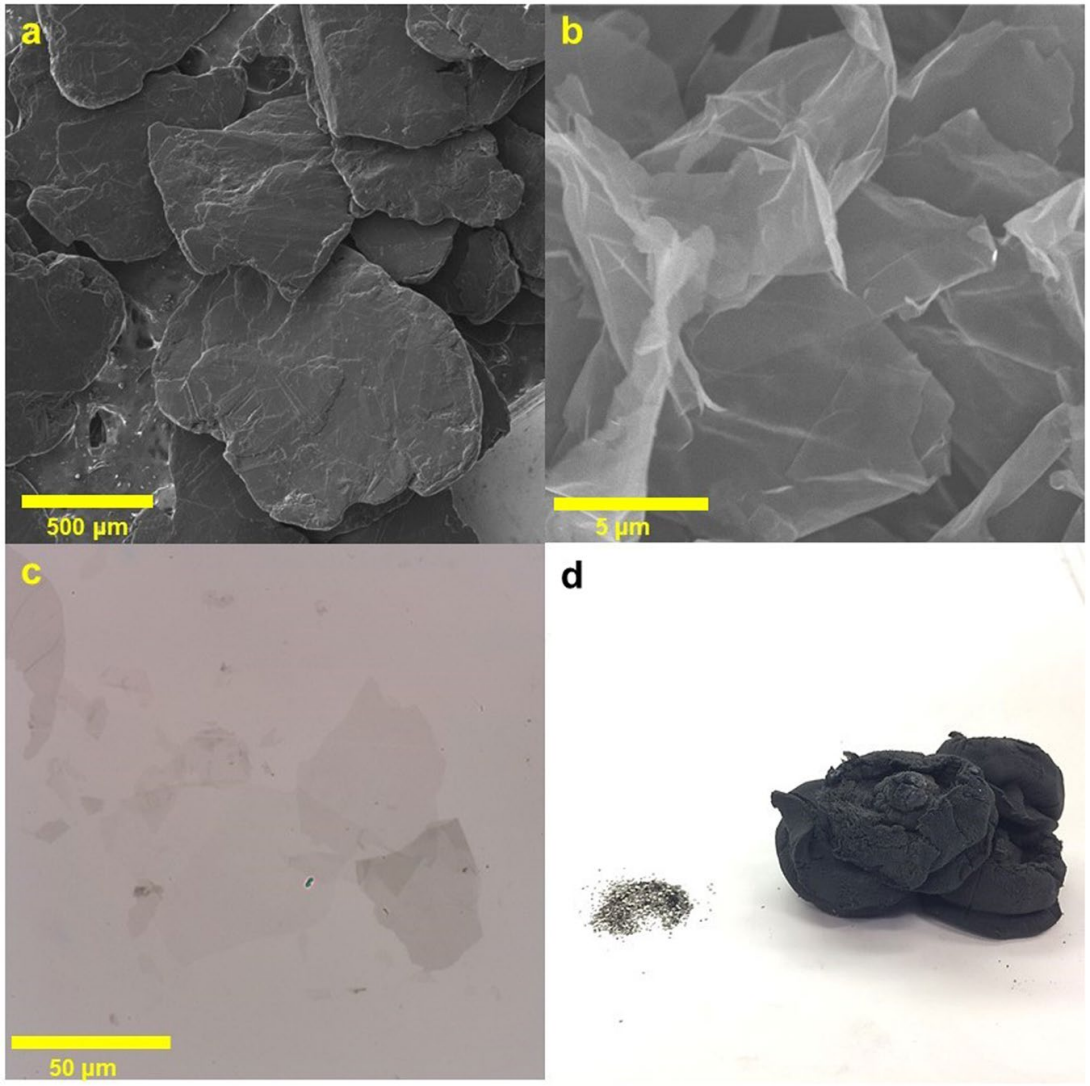
SEM images of (**a**) parent graphite and (**b**) EEG-4 (8000 rpm, 1 hour). (**c**) Bright-field optical microscopy of EEG-3 (1000 rpm, 16 hours). (**d**) Volumetric change of equivalent masses (0.2 g) of parent graphite on left and EEG-4 on right.

In order to probe the morphology of the produced EEG-2 and EEG-4, scanning electron microcopy (SEM) was performed. Figure 3a shows an SEM image of the parent graphite flakes, which are flat and thick with a mean lateral size of over 1000 µm. Immediately after our electrochemical expansion process, the EEG-2 morphology has a paper-like appearance, with a much higher degree of expansion than in prior methods (Fig. S8). After separation and shear mixing, the resulting EEG-4 sheets consistently show a classic graphene morphology with no unexfoliated material present (Figs 3b, S9). One remarkable aspect of this EEG-3 is the high lateral size, which is even observable in optical microscopy images (Figs 3c, S10). The lateral size of EEG-3 was ~40 µm; this is consistent with additional AFM images (Figs S11, 12). We also observe the expansion of graphite in volume; as shown in Fig. 3d, the freeze-dried EEG occupies a much higher volume than the parent graphite flakes. A leftward shift in the graphite (002) peak in the X-ray diffraction (XRD) spectrum implies disordered restacking of graphene (Fig. S13).

### Scaleup

Yield is one of the most critical metrics for scalable production. Note that the field of graphene production has suffered from a lack of standardization on this point. For example, prior efforts have calculated yield as simply the mass liberated from the graphite electrode divided by the original graphite mass^36^. This is somewhat misleading, because after the yield is determined, additional sonication and centrifugation (i.e. separation) is needed to produce graphene such that only a fraction of that material actually becomes few-layer graphene. In contrast, we define our yield as the EEG-1 produced after electrochemical exfoliation and sedimentation, with no additional separation steps that reduce the yield; our characterization data above indicate that all of the as-produced EEG-1 can be considered graphene (rather than only a small fraction as in prior methods). Our prototype batch reactor results in yields of 38% (only 15% if the graphite is not pre-treated with HNO_3_). Below, we show that this number can reach 65% and possibly higher.

**Figure 4:**
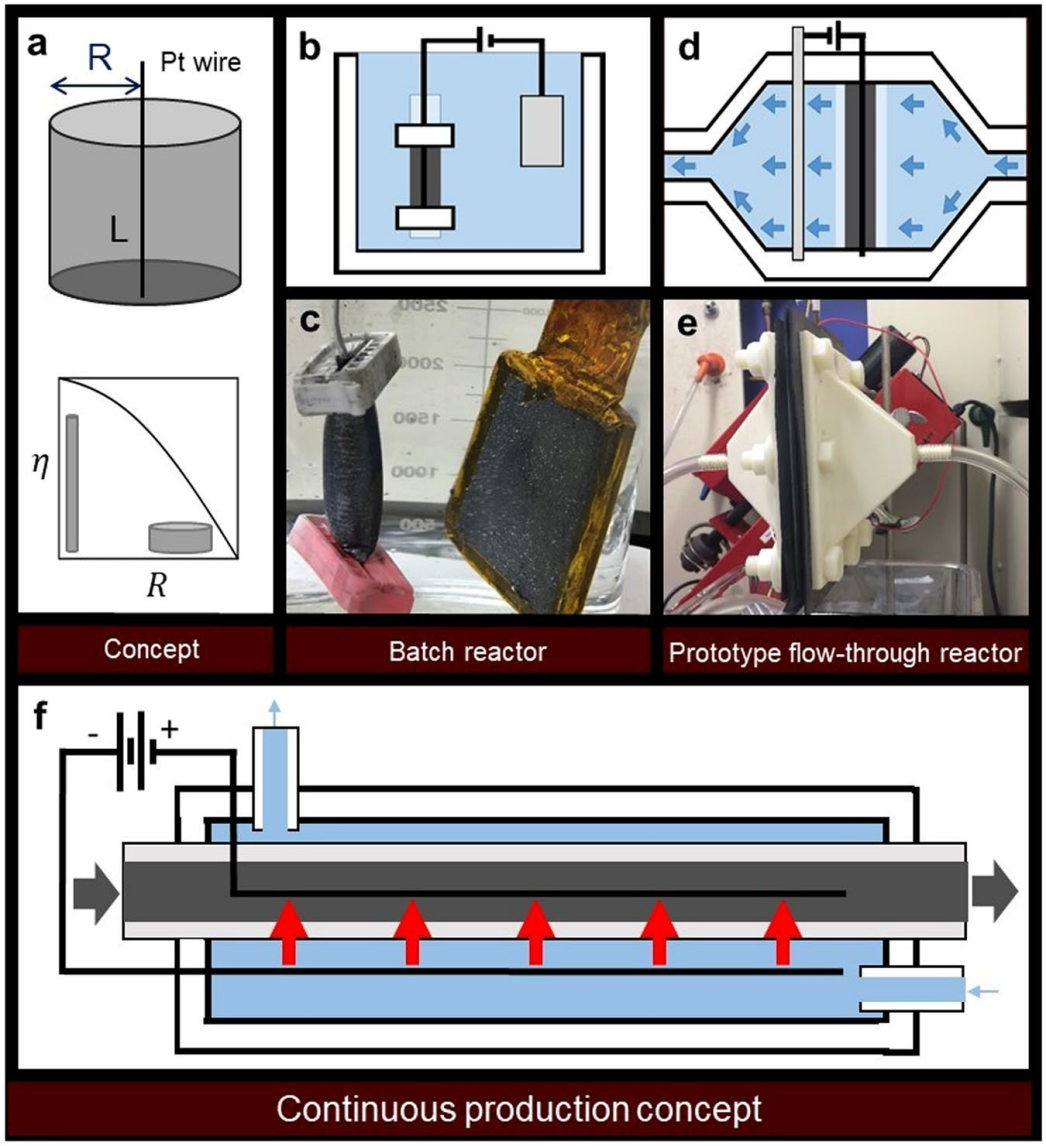
(**a**) Diagram of the conceptual model of the electrochemical exfoliation process, showing that efficiency of cylindrical electrode exfoliation varies inversely with radius R at large R values. (**b**) Diagram and (**c**) picture of prototype batch reactor, with working electrode in an expandable, permeable cylindrical container. (**d**) Diagram and (**e**) picture of prototype reactor for forced electrolyte flow through a tileable 2-D graphite electrode with platinized titanium mesh. (**f**) Schematic for continuous production of EEG in a permeable plug-flow reactor with forced electrolyte flow.

The working electrode (graphite flakes compressed in a permeable container) can be modeled as an electrically conductive cylinder (Fig. 4a) where the compressed graphite electrode has a radius of R and a length of L; this roughly corresponds to our initial reactor setup (Fig. 4b,c). The efficiency (η) of the graphite-to-graphene process may drop below 100% because of transport limitations; η is independent of L because the ion transport occurs chiefly in the radial direction. Two key limitations arise: (i) Radial voltage drop will limit the local potential, affecting ion intercalation and oxidation. We ensure the graphite flakes are sufficiently compressed to maintain electrical connection (Fig. S1**)**, and we assume that the electrical resistance of the Pt wire is negligible. As R increases, the average voltage drop from the Pt wire to any given graphite location increases and the efficiency (η) is lowered. (ii) Similarly, ion diffusion into the interior may be the limiting factor; for isothermal ion diffusion-limited reactions, the efficiency η scales with R^−1^ (regardless of geometry or reaction order) because the Thiele modulus scales linearly with R^45^. Note that this problem of ion diffusion into the interior is exacerbated as graphite is converted to graphene and the tortuosity increases inside the electrode. (One other potential scaleup limitation is the area of the counter electrode, which must scale with the cross sectional area of the working electrode).

With these limitations in mind, the electrochemical reactor was redesigned (Fig. 4d,e) to increase the surface-to-volume ratio of the electrode (to reduce ion diffusion limitations), increasing both working electrode and counter electrode area, and decrease electrode separation. To ensure electrolyte transport into the interior of the graphite electrode, a forced electrolyte convection system was fabricated using thermoplastic additive manufacturing. A platinized titanium mesh is inserted into the graphite electrode, which is held in place by filters in an acrylic frame. The counter electrode is a steel grate. This reactor design has the benefit of being infinitely tileable in the plane of the reactor, implying that efficiency is unchanged by scaleup. Utilizing these methods, yield was increased to 65%; this increase indicates that electrolyte diffusion limitations play a major role.

In addition to batch processing, a continuous electrochemical reaction scheme is proposed in which flake graphite is conveyed through a screw reactor and electrolyte is forced under pressure through a porous pipe or ridged mesh (Fig. 4f**)**. These graphite flakes would be in contact with an in-line screw conveyer that doubles as the working electrode, and are continually electrochemically exfoliated as the graphite moves through the reactor. The average exfoliation residence time τ in the continuous reactor is controlled by the graphite flow rate Q and the geometry (area A, length L) of the reactor as τ = AL/Q. This is directly scalable. The flowing graphite must be at a high enough concentration to ensure electrical contact, such that the flow is a dense slurry. This is only one embodiment of the possible continuous electrochemical reactors that uses this compression technique, and many potential designs exist that could lead to efficient continuous graphene production. Additional scaleup factors include: Number, geometry, and material of electrodes and current collectors; parent graphite particle size, and electrolyte distribution systems.

## Conclusions

We successfully demonstrated that graphite powder can be electrochemically exfoliated into graphene in a permeable and expandable container as a monolithic working electrode. Raman spectra and other characterization data suggest a high yield of graphene with large sheet sizes (>30 µm). In addition, various sources of graphite flakes/powder can be utilized as the parent material using our approach. We also demonstrated design principles for graphene reactors, including geometric effects on efficiency and electrolyte transport; these design principles indicate that scaleup of continuous graphene production can be accomplished without compromising yield or quality.

## Materials and Methods

The datasets generated during and/or analyzed during the current study are available from the corresponding author on reasonable request.

### Electrochemical expansion

5 g of flake graphite (Sigma-Aldrich) is compacted in dialysis tubing (cellulose membrane, average flat width 25 mm) with a platinum wire inside as the current collector. (Note that β-lead oxide is also effective well as a current collector on the working electrode.) The dialysis tubing is clipped on both sides to maintain the integrity. This assembly serves as the cathode. The compact cathode is immersed in 3 L of 0.1 M ammonium sulfate ((NH_4_)_2_SO_4_) aqueous solution as electrolyte. A flat piece (~10 cm × 10 cm) of graphite foil serves as the anode. The cathode and anode are connected to a power supply. 10 volts are applied to the platinum electrode immersed in dialysis bag filled with graphite flakes during electrochemical expansion, with a compliance current of ~0.5 A. Electrochemical expansion time varies from 1–24 hours. The EEG is then washed via centrifugation with distilled water for five times. The EEG can be distinguished from flake graphite by its matte black color and high porosity. The yield is calculated by freeze dried weight of EEG-2 collected over the initial weight of graphite placed in the tubing. After washing, the supernatant was transferred into a 400 mL Erlenmeyer flask, and the flask was filled with 300 mL of DI water. After settling for 10 minutes, the supernatant was transferred into a beaker, labeled EEG-1 for further characterization and treatments. The sediment was unexpanded graphite.

To obtain free nanosheets, this EEG-1 is shear mixed in DI water at 1000 or 8000 rpm for 1 hour. The EEG that is collected is characterized by optical microscopy, SEM, TGA, XRD, XPS. The EEG graphene that is collected was then characterized by AFM, SEM, TEM, TGA, and Raman Spectroscopy.

### Characterization procedures

Aqueous EEG-1 was flash frozen using liquid nitrogen and freeze dried for roughly 48 hours in Labconco FreeZone benchtop freeze dryer to obtain EEG-2. Surface morphology of EEG-2 and EEG-4 was imaged with a Multimode scanning probe microscope (AFM) (Bruker Dimension Icon) operated in tapping mode and a scanning electron microscope (SEM) (JEOL JSM-7500F instrument) at 5 kV accelerating voltage. The EEG-1 and EEG-3 was dropcasted on freshly cleaved mica surface for AFM imaging. The EEG-2 and EEG-4 was pressed on carbon tape for SEM imaging. X-ray photoelectron spectroscopy (XPS) was conducted using an Omicron XPS system with Mg X-ray source and the data analysis was performed using CasaXPS software (Casa Software, Ltd.). The EEG-2 was pressed on carbon tape for XPS. The Raman spectra were obtained using a ThermoScientific DXR Raman confocal microscope with 900 g/mm gratings for excitation lasers with a wavelength of 532 nm. The EEG-2 and EEG-4 was pressed on a piece of glass slide for Raman spectroscopy. For bright-field optical microscopy, the EEG-1 and EEG-3 sheets were suspended in DI water and deposited on a glass slide. X-ray diffraction (XRD) patterns were collected on Bruker D8 Discover diffractometer fitted with LynxEye detector in a Bragg Brentano geometry at 40 kV, 40 mA with CuKα (λ: 1.5418 Å) radiation. The EEG-2 was pressed on a piece of glass slide to obtain the XRD patterns with a scan rate of 1 s per step and a step size of 0.02°. Thermogravimetric Analysis (TGA) was performed in a TA Instruments Q50 TGA to determine the mass loss of the EEG-4. ~10 mg of each sample was heated up in a ceramic pan from room temperature to 1000 °C at a rate of 1 °C/min in air. The electrical resistance of the EEG-5 was measured using the four-point probe method. The four-point probe head (Signatone, SP4-40045TBY) was mounted on a resistivity measurement stand (Signatone, Model 302). The spacing between the probe tips was 1.5875 mm. The current was passed to the sample through the outer probes using a Keithley 6221 AC and DC current source. A Keithley 2000 multimeter was used to measure the voltage across the sample. The sheet resistance and electrical conductivity of the samples were calculated using the measured voltage values.

## Electronic supplementary material

Supplementary Information
