# Supplementary material for: High-yield scalable graphene nanosheet production from compressed graphite using electrochemical exfoliation
Source: Sci Rep. 2018 Sep 28;8:14525. doi: 10.1038/s41598-018-32741-3 (PMC6162260; doi:10.1038/s41598-018-32741-3)
Supplement: Supplementary file 1 — Supplementary Information [file 41598_2018_32741_MOESM1_ESM.pdf]

High-yield scalable graphene nanosheet production from compressed graphite using electrochemical exfoliation

Thomas C. Achee,[1] Wanmei Sun,[1] Joshua T. Hope,[1] Samuel G. Quitzau,[1] Charles B. Sweeney,[1,2] Smit A. Shah,[1] Touseef Habib,[1] Micah J. Green[1,2*]

[1]Artie McFerrin Department of Chemical Engineering, Texas A&M University, College Station, TX
[2]Department of Materials Science and Engineering, Texas A&M University, College Station, TX

*corresponding author: micah.green@tamu.edu

Supporting Information

Measurements of graphite resistivity as a function of pressure

This experiment shows the intuitive drop in resistivity as applied pressure leads to a decrease in the tortuosity of the electronic path through the graphite. This high resistivity in the low-pressure regime implies a steeper voltage drop and thus highly inefficient electrochemical exfoliation in uncompressed systems. (These data were collected without electrolyte.) The data in **Figure S1** shows that resistivity is a strong function of pressure.

Method: Using a lab balance to measure force applied, a known volume of flake graphite (1 cm^3) was placed under varying amounts of pressure. The area the force was applied over, A, is known (1 cm^2). This allows the calculation of pressure via its definition, P = F/A. The resistance of the volume was measured with a multimeter between two copper contacts. Resistivity was calculated as $\rho = Rl/A$. A correction had to be made to account for the compression of the graphite, changing l. The resistivity of the copper contacts was assumed to be negligible due to their short length.

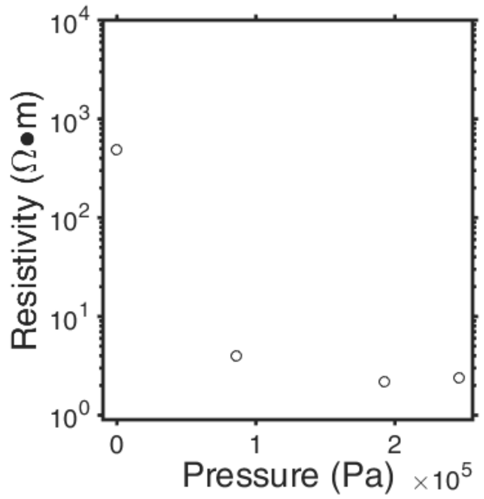

Figure S1. Variation of resistivity of flake graphite vs applied pressure

HNO_3 treatment of graphite

We used HNO_3 to convert hydrophobic graphite to hydrophilic graphite by adding oxygen-containing groups to any pre-existing defect sites; a similar method was effective in functionalizing multi-walled carbon nanotubes.[1] As shown in **Figure S2a,** upon addition of both treated and untreated graphite flakes to DI water, most of the untreated graphite flakes float on water, while most of the HNO_3-treated graphite flakes precipitate in water. This pronounced difference in initial wetting demonstrates conversion of the originally hydrophobic graphite flakes to hydrophilic graphite flakes by the HNO_3 treatment.

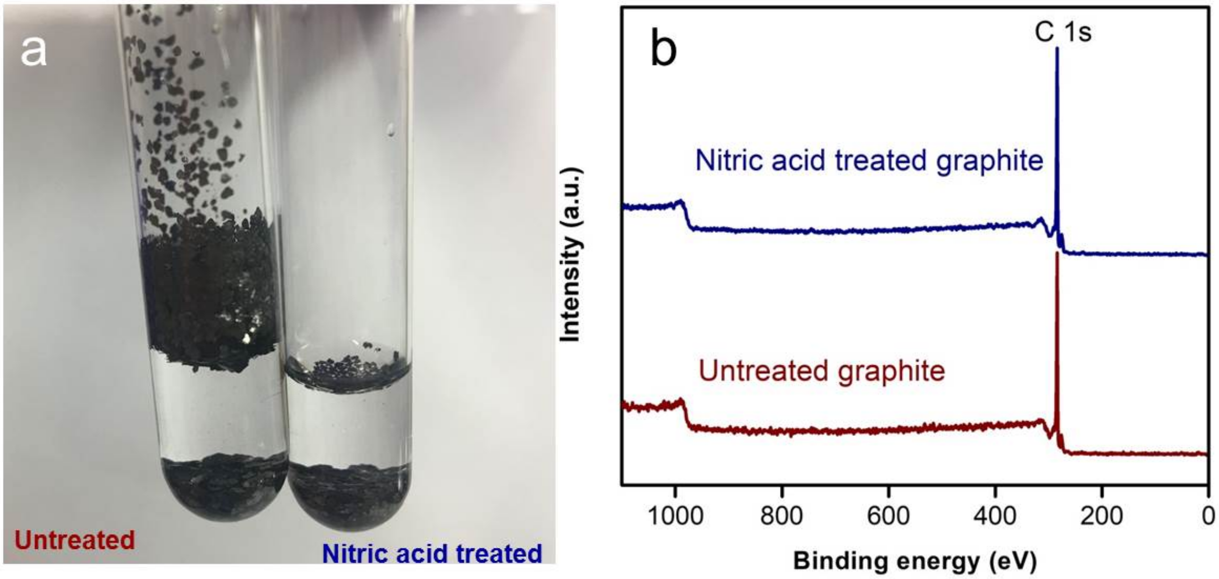

Figure S2. (a) Comparison of untreated (left) and HNO_3-treated (right) graphite flakes in DI water. (b) XPS survey of HNO_3-treated graphite and untreated graphite.

HNO_3 treated graphite flakes precipitate in water indicating hydrophilicity.[1] A greater number of untreated (hydrophobic) graphite flakes are not wetted by the water in the vial depicted on the left due to the higher interfacial tension between the water and untreated material as compared to the interfacial tension between the water and the treated material. However, the difference between the oxygen content of HNO_3-treated graphite and untreated graphite is not pronounced in XPS survey in **Figure S2b**.

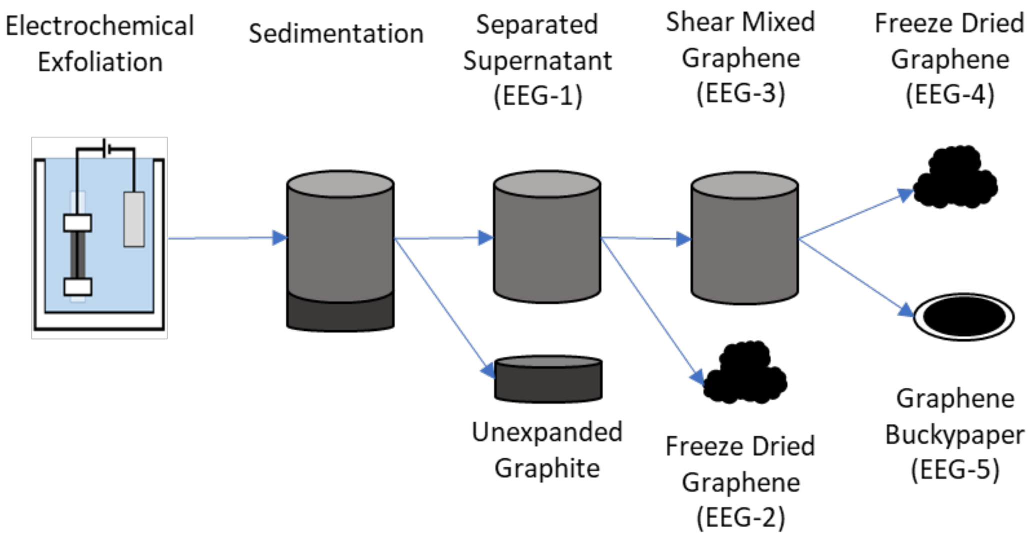

Figure S3. Numerous characterization methods were used at various steps in the process to verify that the produced material was indeed graphene.

Raman spectra

To study the effect of shear mixing, we varied shear mixing rate and time for graphene samples that have been electrochemically exfoliated for 24 hours. No pronounced difference in Raman spectra exists for those samples. However, the sample in **Figure 2e** has been exposed to only 4 hours of the electrochemical exfoliation process, resulting in less overall oxidation than the 24-hour samples in **Figure S4**; this is the main reason for the difference in the Raman spectra. Even so, there is spot-to-spot variability (due to localized oxidation) in the Raman results for these 4-hour samples.

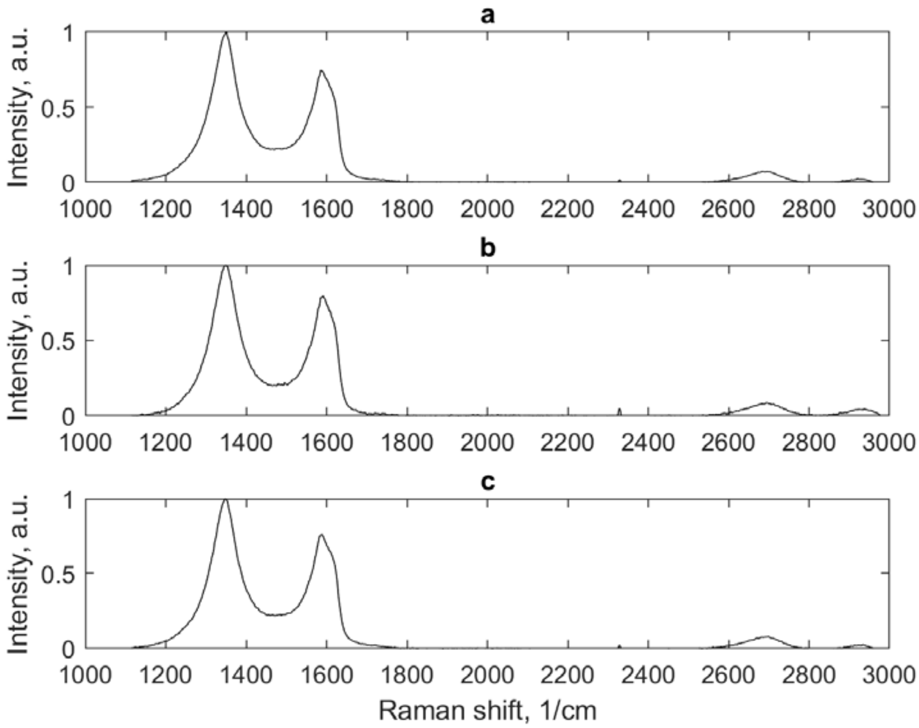

Figure S4. Raman spectroscopy of different EEG-2 and EEG-4 (all of them have been electrochemically exfoliated for 24 hours with different shear mixing rate and time) samples. (a) EEG-2, (b) EEG-4 shear mixed at 1000 rpm for 24 hours, (c) EEG-4 shear mixed at 8000 rpm for 24 hours.

Thermogravimetric analysis

Thermogravimetric analysis (TGA) allows us to compare the degree of oxidation between conventional graphene oxide (GO) from Hummer's method. Prior work has shown that $T_{1/2}$, the temperature at which half of the material is removed, is related to the degree of oxidation.[2,3] We collected the TGA data of different graphite, EEG-2, and graphene oxide samples in air flow (**Figure S5**). The $T_{1/2}$ sequence of those samples are as follows: parent graphite flakes > EEG-2 > GO. This observation implies the degree of oxidation of different samples are in the following order: GO > EEG-2 > parent graphite flakes.

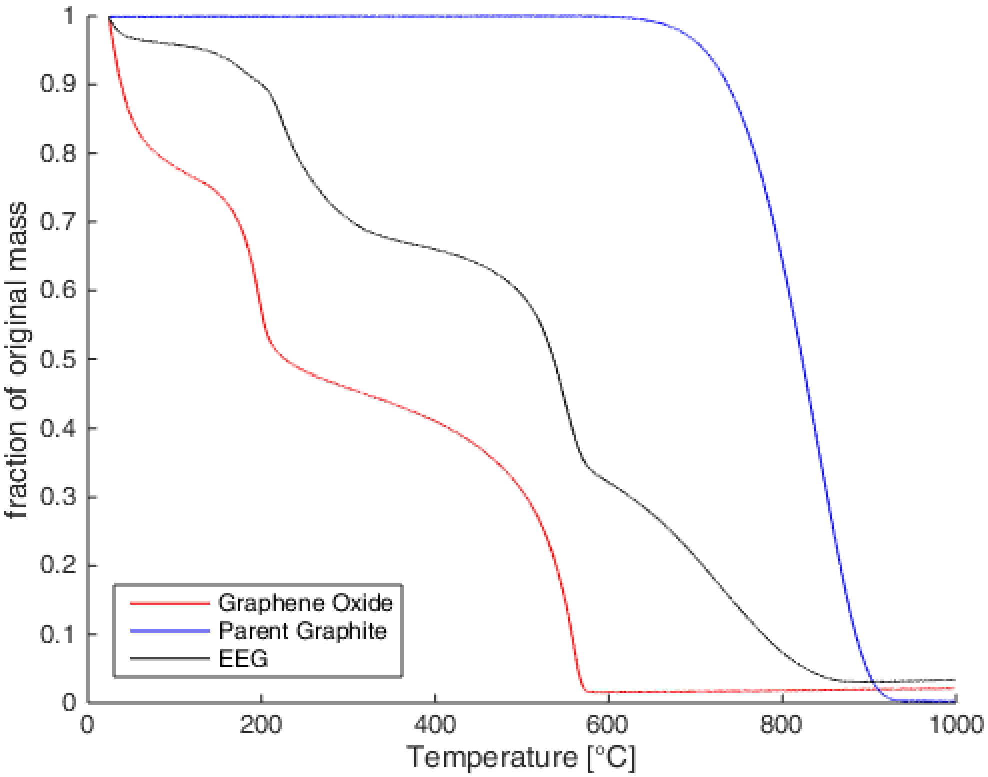

Figure S5. The TGA signature of EEG-2 lies between that of the parent graphite and GO from Hummers' method.

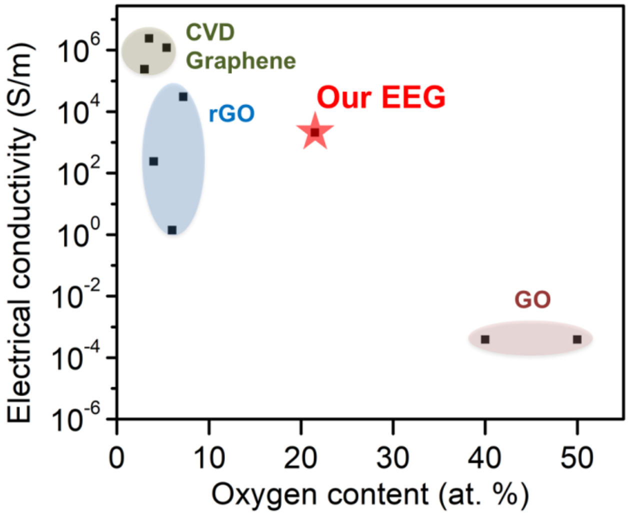

Figure S6. Electrical conductivity vs. oxygen content for EEG-5 compared with films composed of other graphene materials. Note: all the electrical conductivities of CVD graphene are calculated based on the sheet resistance and transparence. We estimate that one single layer of graphene would show an optical transparency of 97.7% and have a thickness of ~0.345 nm. For the CVD graphene citations with a transparency of ~95%, we estimate that this CVD graphene has two layers of carbon atoms, whose thickness is ~0.7 nm.[2-8]

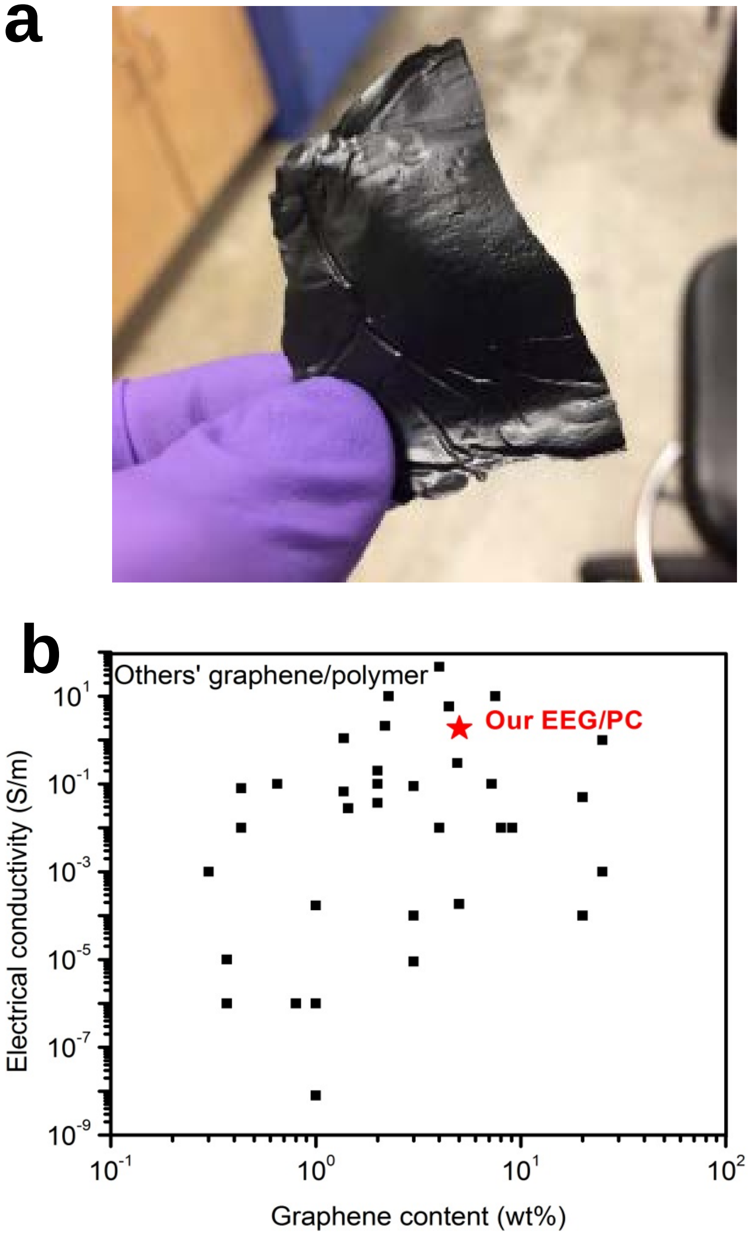

Figure S7. (a) Photograph of 5 wt% EEG-4 in polycarbonate composite. (b) Summaries of the electrical conductivity of graphene/polymer composites (prior work summarized in Parviz *et al*.)[4]

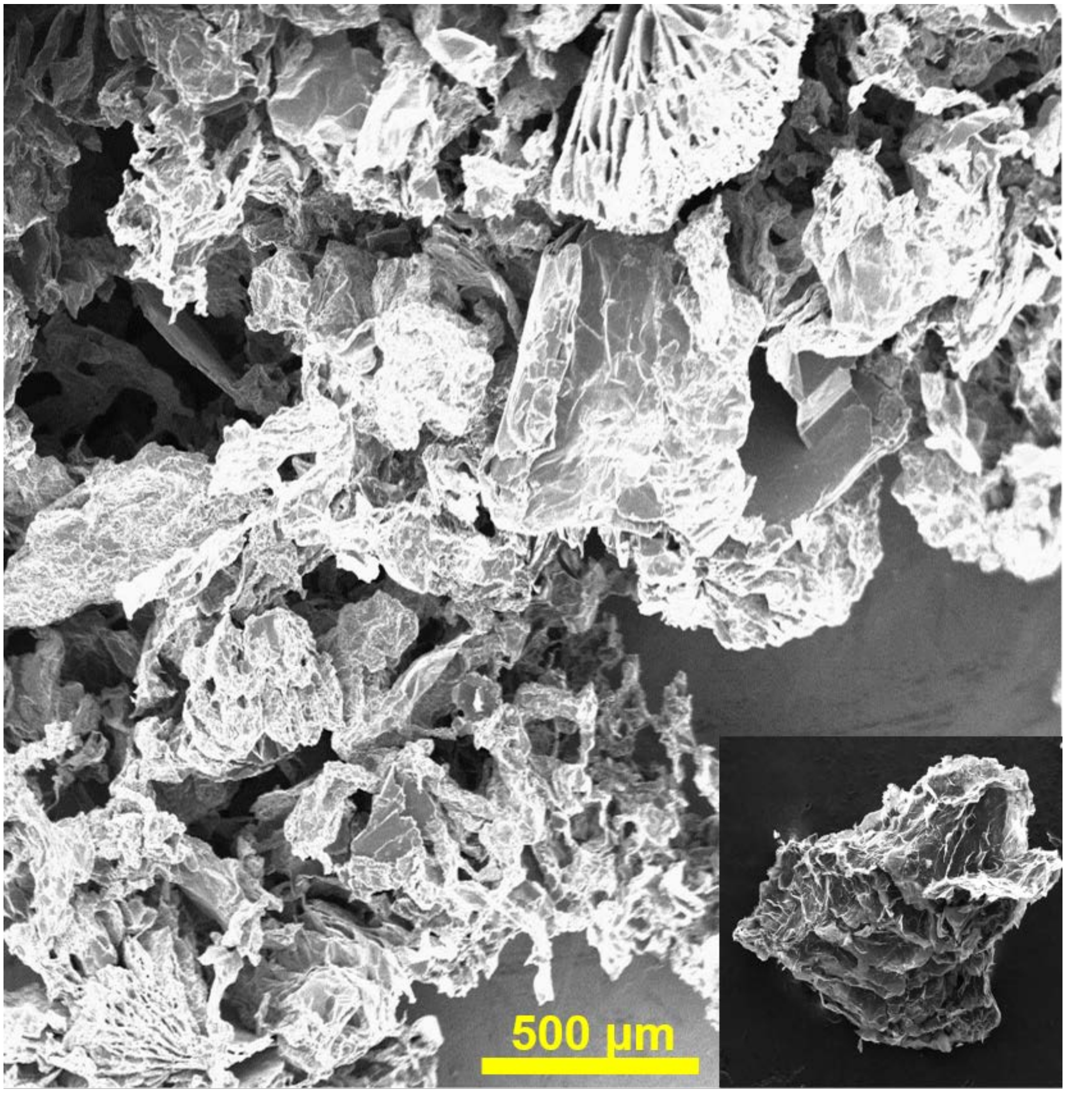

Figure S8. SEM micrographs (both on the same scale) comparing EEG from published methods[5] (right) to EEG-2 from our method (left). Both samples were imaged before any secondary mechanical exfoliation step. Note the higher degree of expansion and thinner, sheet-like structures in the left image. The scale bar is the same for both images.

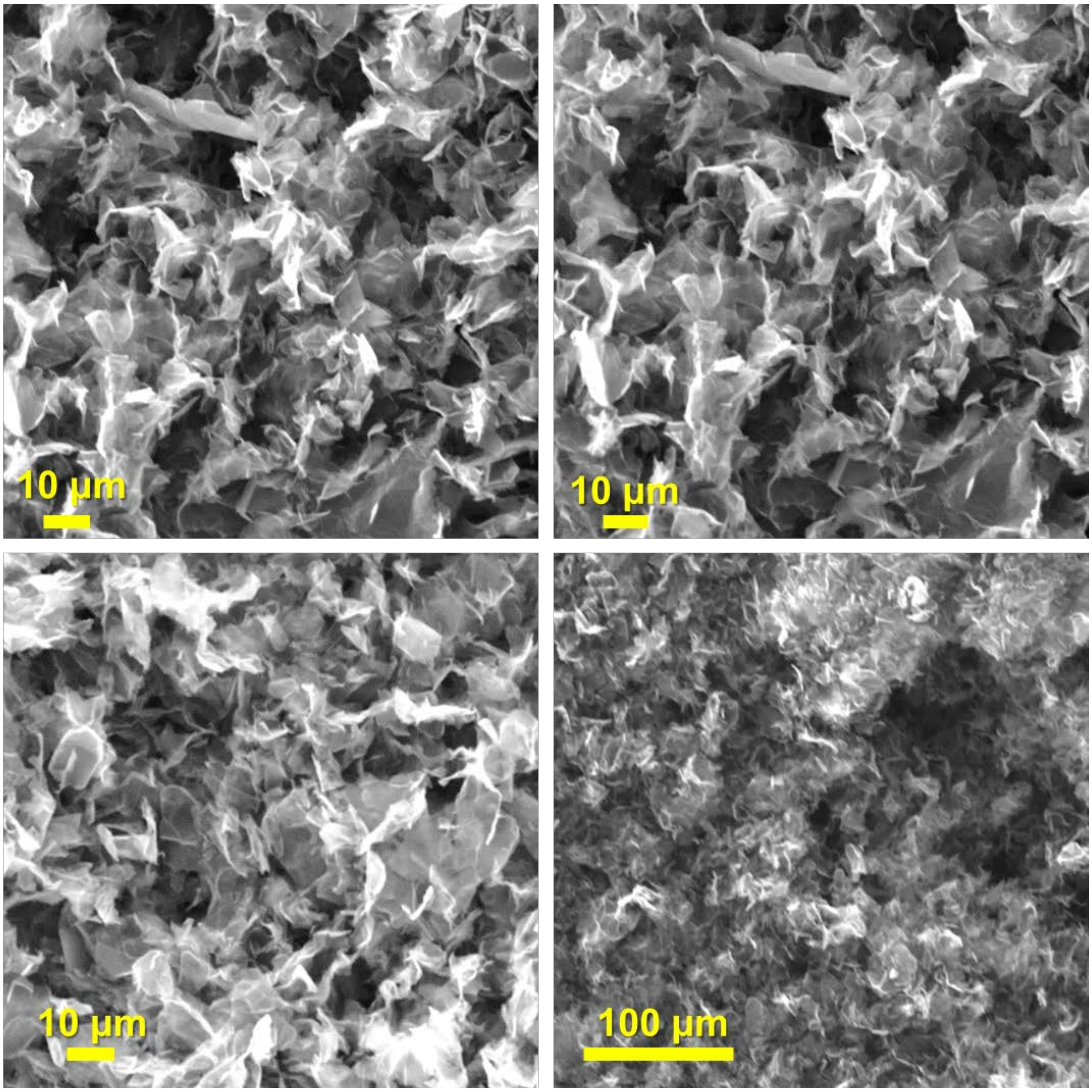

Figure S9. Four SEM images of EEG-4 (8000 rpm, 1 hour) showing the absence of parent material in the final product, and high degree of expansion.

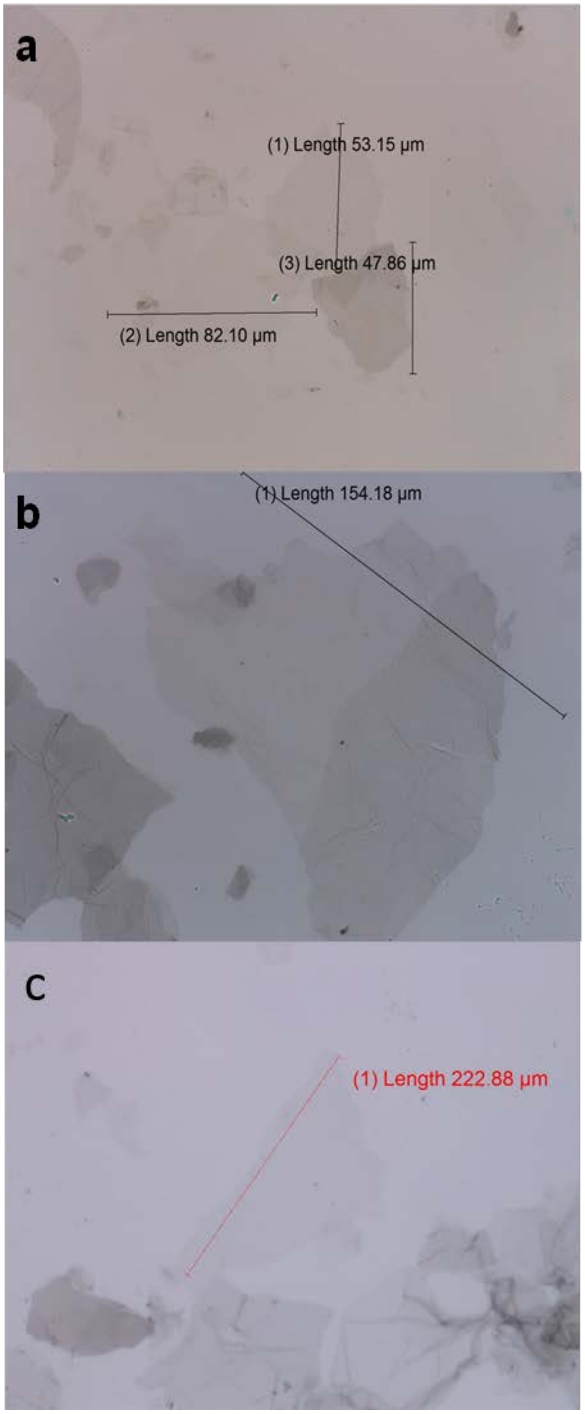

Figure S10. Optical microscope images of shear mixed (24 hour, 1000 rpm) EEG-4, illustrating the large lateral size that can be achieved through electrochemical exfoliation.

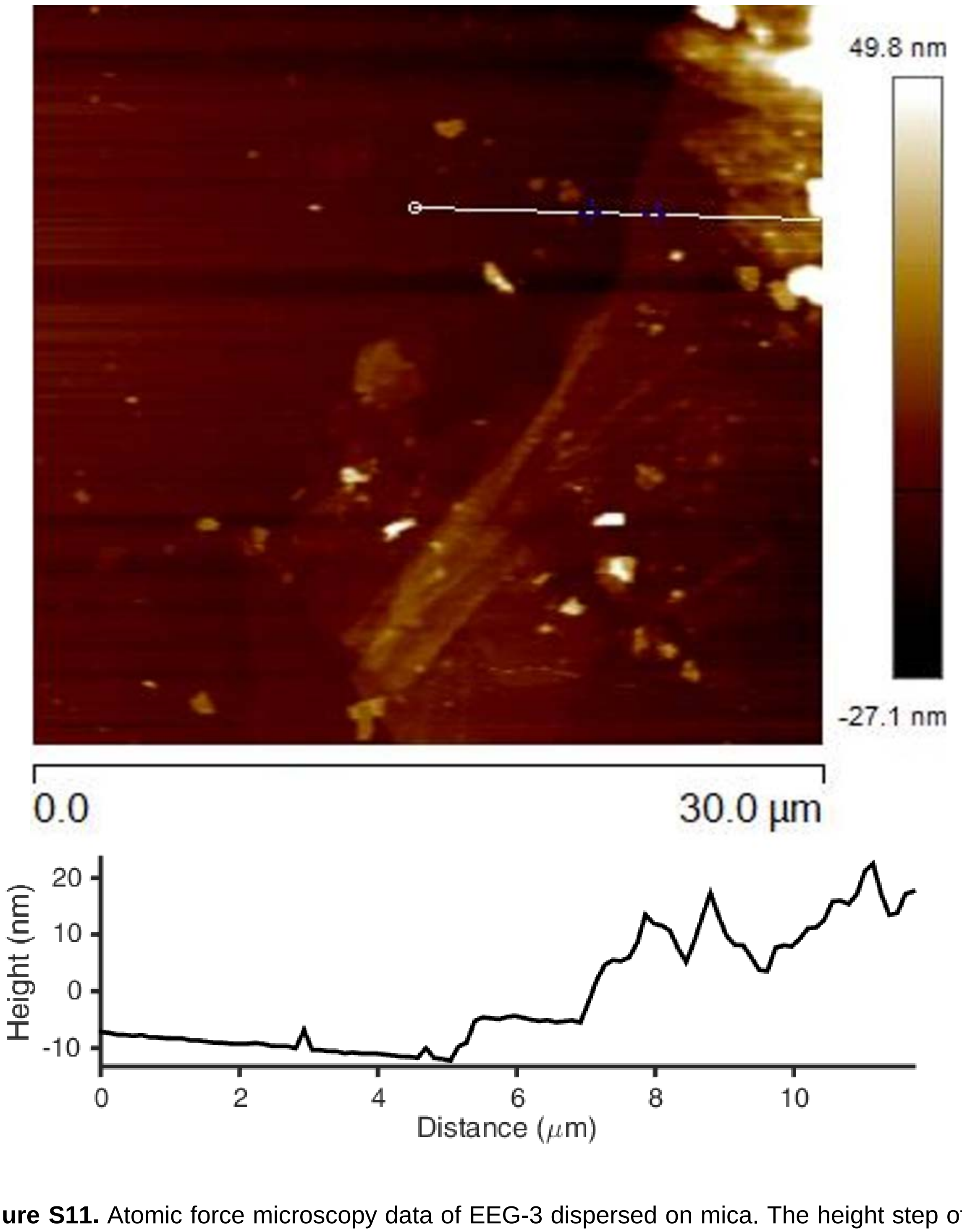

Figure S11. Atomic force microscopy data of EEG-3 dispersed on mica. The height step of this sheet is 10nm. Note the folded morphology that is common for electrochemically exfoliated samples.

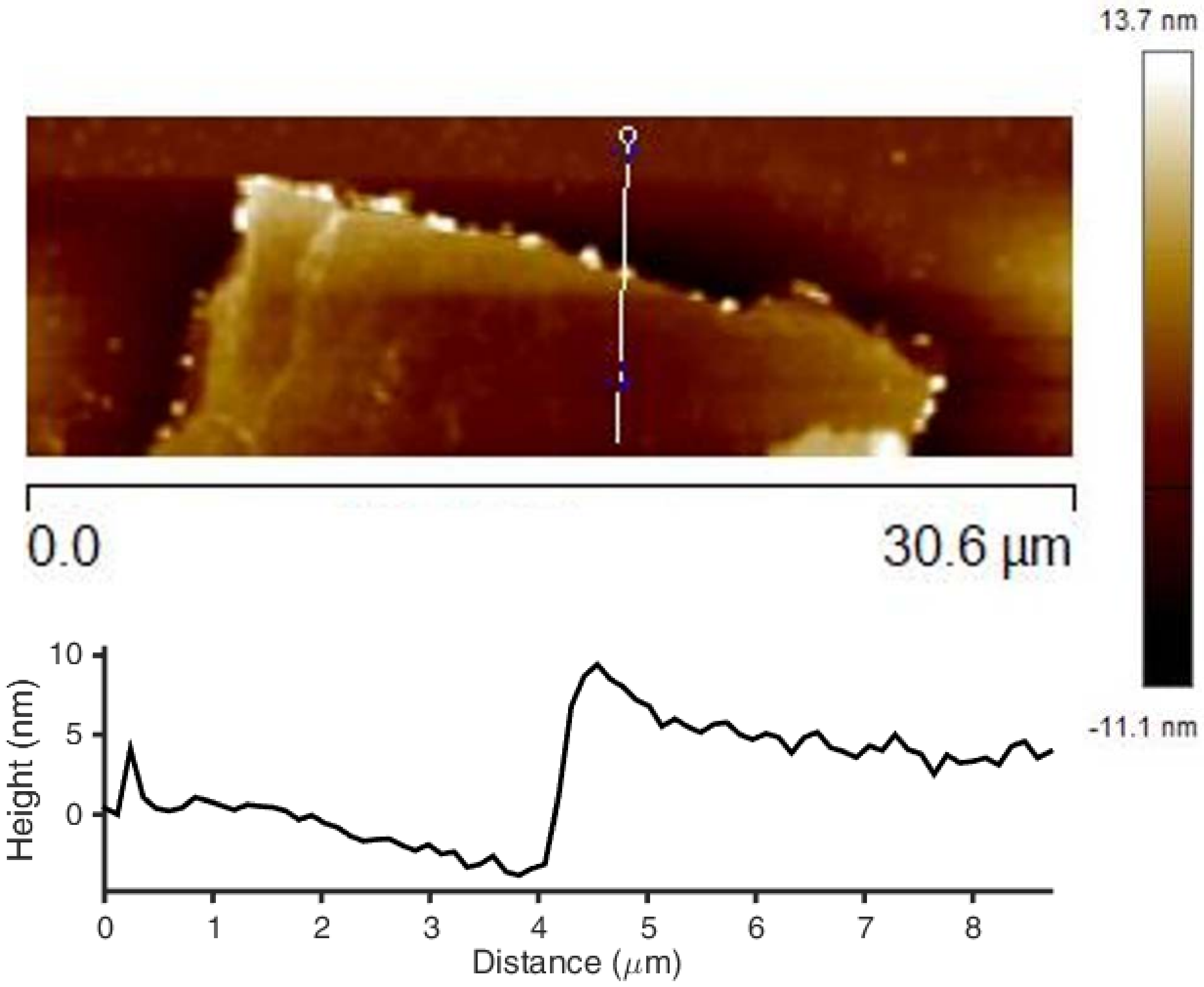

Figure S12. Atomic force microscopy data of EEG-3 dispersed on mica. The height difference between the basal plane of the sheet and the substrate is 3 nm.

X-ray diffraction

We also used X-ray diffraction (XRD) to track the expansion of graphite flakes during the electrochemical expansion process as a function of time (**Figure S13)**. The resulting data show that the 2θ peak for (002) shifts from 26.7 ° to 26.6 ° after 24 hours of electrochemical expansion, suggesting the d-spacing has increased from 0.331 to 0.355 nm. The increased d-spacing further confirms the existence of graphene after the electrochemical expansion.

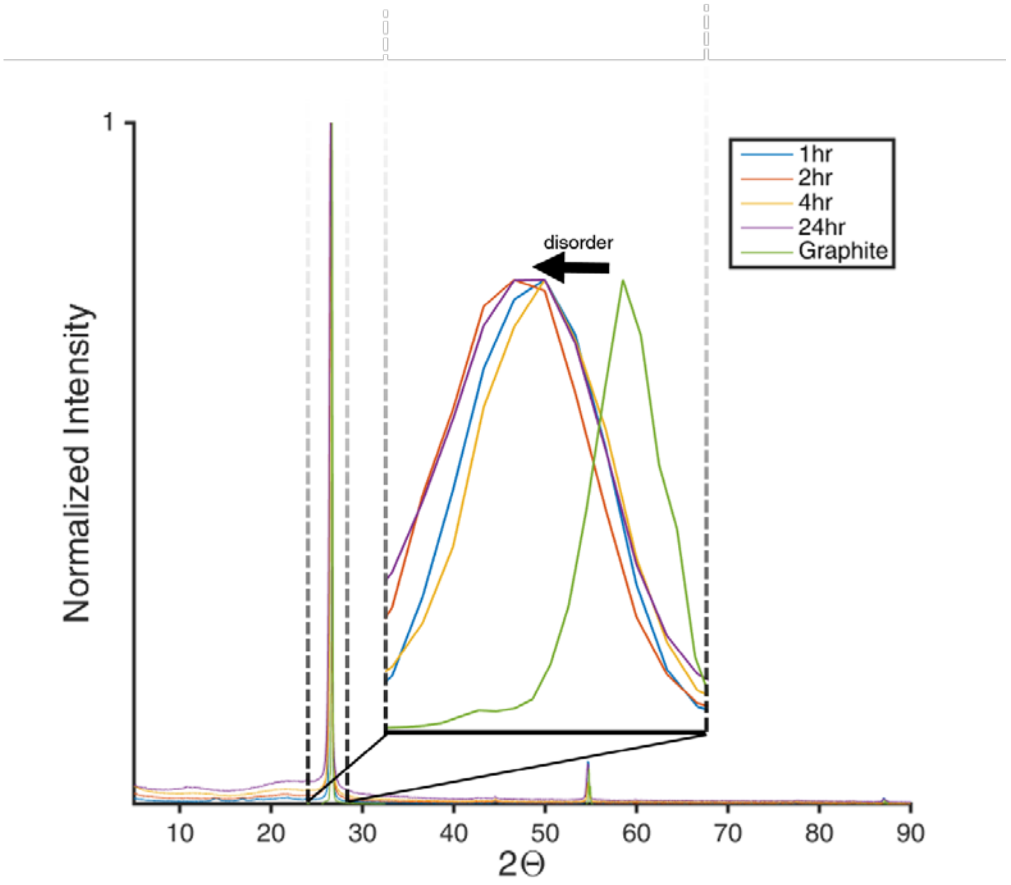

Figure S13. XRD spectrum of EEG-2 as a function of electrochemical processing time. Note the leftward shift of the graphite (002) peak and the broadening of the peaks caused by the disordering of the graphite lattice.

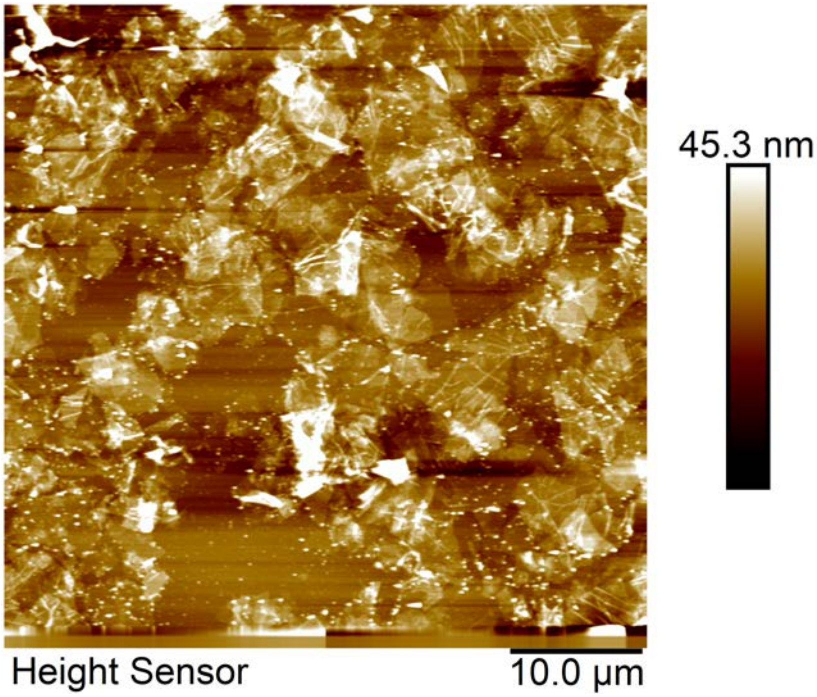

Figure S14. AFM image of EEG-3 from the most recent reactor.

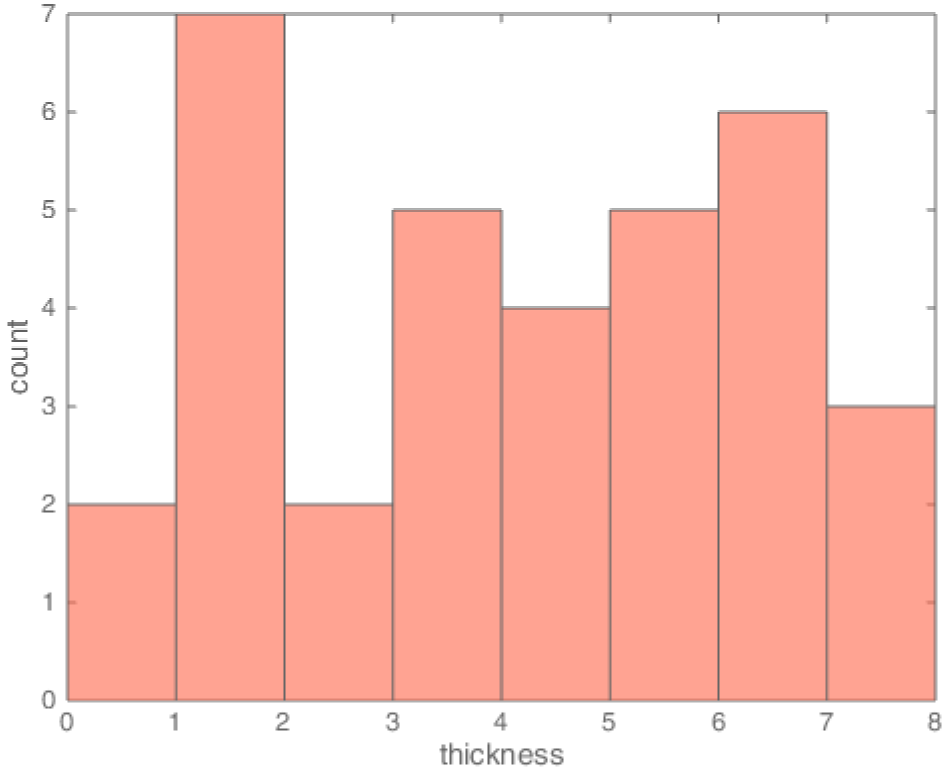

Figure S15. Thickness distribution of EEG-3 from AFM image in Figure S13. The unit of thickness is nm.

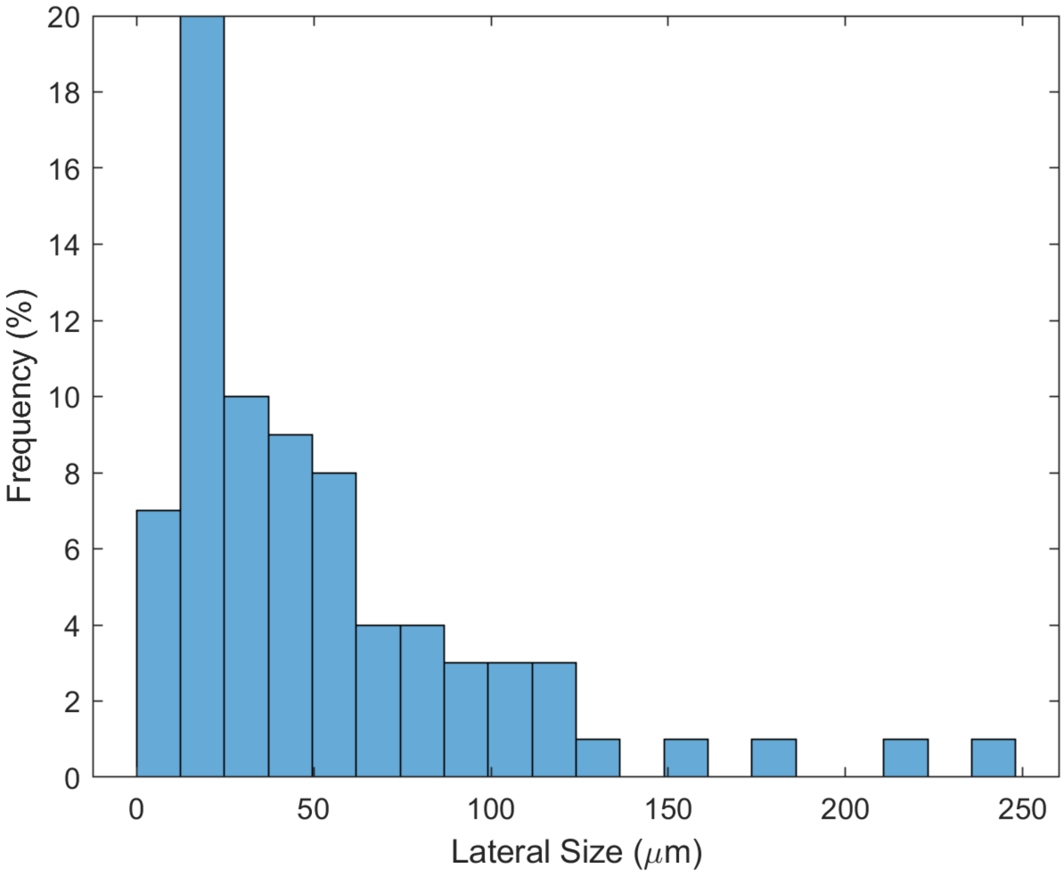

Figure S16. Lateral size distribution of EEG-1 from optical images.

REFERENCES

(1) Datsyuk, V.; Kalyva, M.; Papagelis, K.; Parthenios, J.; Tasis, D.; Siokou, A.; Kallitsis, I.; Galiotis, C. Chemical oxidation of multiwalled carbon nanotubes. *Carbon* **2008**, *46*, 833-840.

(2) Buzaglo, M.; Bar, I. P.; Varenik, M.; Shunak, L.; Pevzner, S.; Regev, O. Graphite-to-Graphene: Total Conversion. *Advanced Materials* **2017**, *29*, 1603528-n/a.

(3) Shtein, M.; Pri-Bar, I.; Varenik, M.; Regev, O. Characterization of Graphene-Nanoplatelets Structure via Thermogravimetry. *Analytical Chemistry* **2015**, *87*, 4076-4080.

(4) Parviz, D.; Irin, F.; Shah, S. A.; Das, S.; Sweeney, C. B.; Green, M. J. Challenges in Liquid-Phase Exfoliation, Processing, and Assembly of Pristine Graphene. *Advanced Materials* **2016**, *28*, 8796-8818.

(5) Parvez, K.; Wu, Z.-S.; Li, R.; Liu, X.; Graf, R.; Feng, X.; Müllen, K. Exfoliation of Graphite into Graphene in Aqueous Solutions of Inorganic Salts. *Journal of the American Chemical Society* **2014**, *136*, 6083-6091.
